# Supplementary material for: CANTAO: guiding clustering and annotation in single-cell RNA sequencing using average overlap
Source: Mol Syst Biol. 2025 Dec 8;22(3):461–75. doi: 10.1038/s44320-025-00176-4 (PMC12954110; doi:10.1038/s44320-025-00176-4)
Supplement: Supplementary file 1 — Appendix [file 44320_2025_176_MOESM1_ESM.pdf]

APPENDIX

CANTAO: GUIDING CLUSTERING AND ANNOTATION IN SINGLE-CELL RNA SEQUENCING USING AVERAGE OVERLAP

Christopher Thai[1,2], Amartya Singh[1,2], Daniel Herranz[1,3,4, #], Hossein Khiabanian[1,2,5,^,#]

Affiliations
[1] Rutgers Cancer Institute, Rutgers University, New Brunswick, NJ 08901, USA
[2] Center for Systems and Computational Biology, Rutgers Cancer Institute, Rutgers University, New Brunswick, NJ 08901, USA
[3] Department of Pharmacology, Rutgers Robert Wood Johnson Medical School, Rutgers University, Piscataway, NJ 08854, USA
[4] Department of Pediatrics, Rutgers Robert Wood Johnson Medical School, Rutgers University, New Brunswick, NJ 08901, USA
[5] Department of Pathology and Laboratory Medicine, Rutgers Robert Wood Johnson Medical School, Rutgers University, New Brunswick, NJ 08901, USA
[^] Current address: Regeneron Genetics Center, Regeneron Pharmaceuticals, Tarrytown, NY 10591, USA
[#] Correspondence to: dh710@cinj.rutgers.edu or h.khiabanian@rutgers.edu

Table of Contents

Appendix Figure S1……………………………………………………………………………1
Appendix Figure S2……………………………………………………………………………2
Appendix Figure S3……………………………………………………………………………3
Appendix Figure S4……………………………………………………………………………4
Appendix Figure S5……………………………………………………………………………5
Appendix Figure S6……………………………………………………………………………6
Appendix Figure S7……………………………………………………………………………7
Appendix Figure S8……………………………………………………………………………8
Appendix Figure S9……………………………………………………………………………9
Appendix Figure S10…………………………………………………………………………10
Appendix Figure S11…………………………………………………………………………11
Appendix Figure S12…………………………………………………………………………12
Appendix Figure S13…………………………………………………………………………13
Appendix Figure S14…………………………………………………………………………14

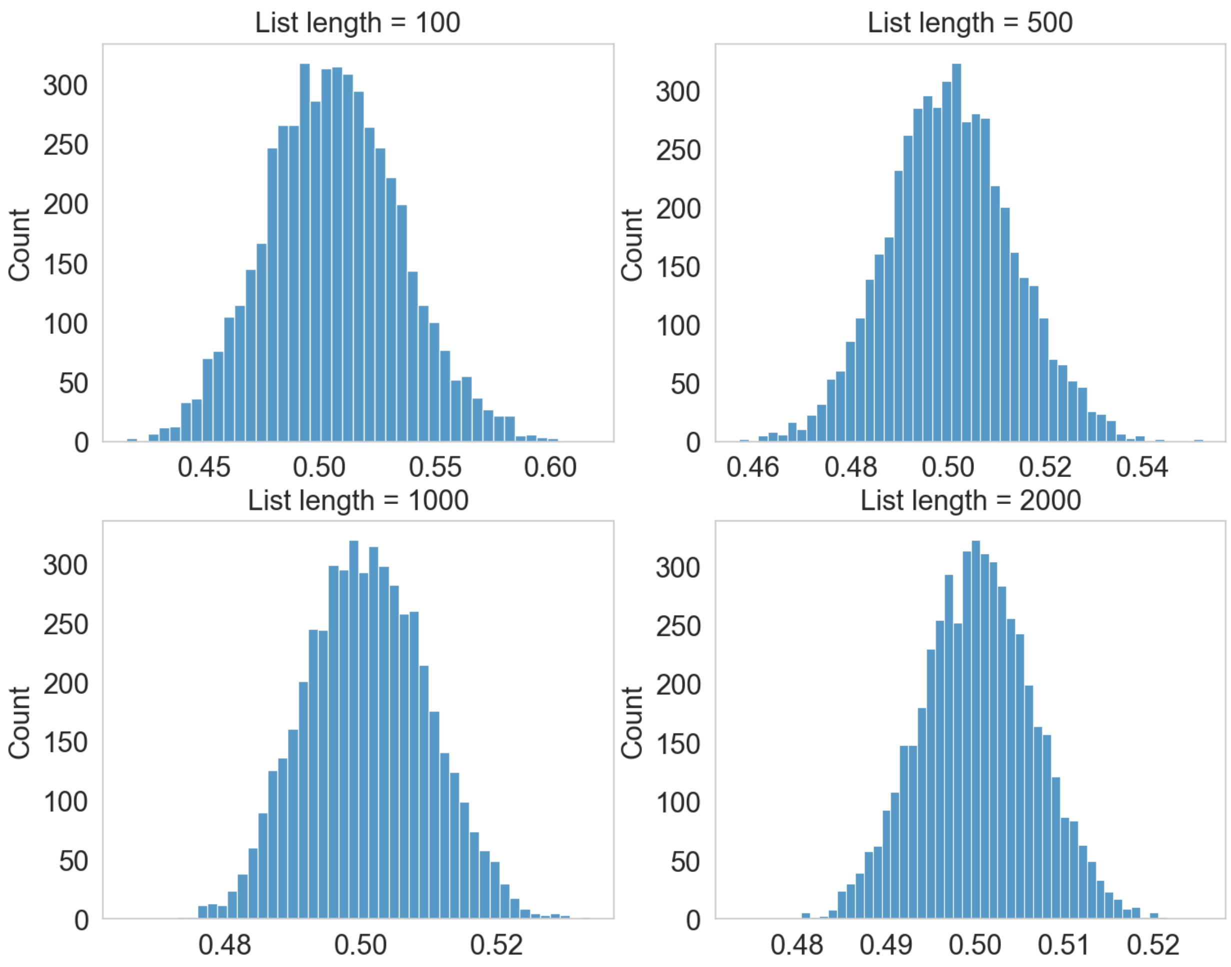

Appendix Figure S1. Distributions of pair-wise average overlap scores on randomly shuffled lists.

Across 2,000 iterations, randomly shuffling two ranked lists that contain the same set of elements yields average overlap distances that closely follow a normal distribution with a mean of 0.5 and a variance that is inversely correlated with the length of the list.

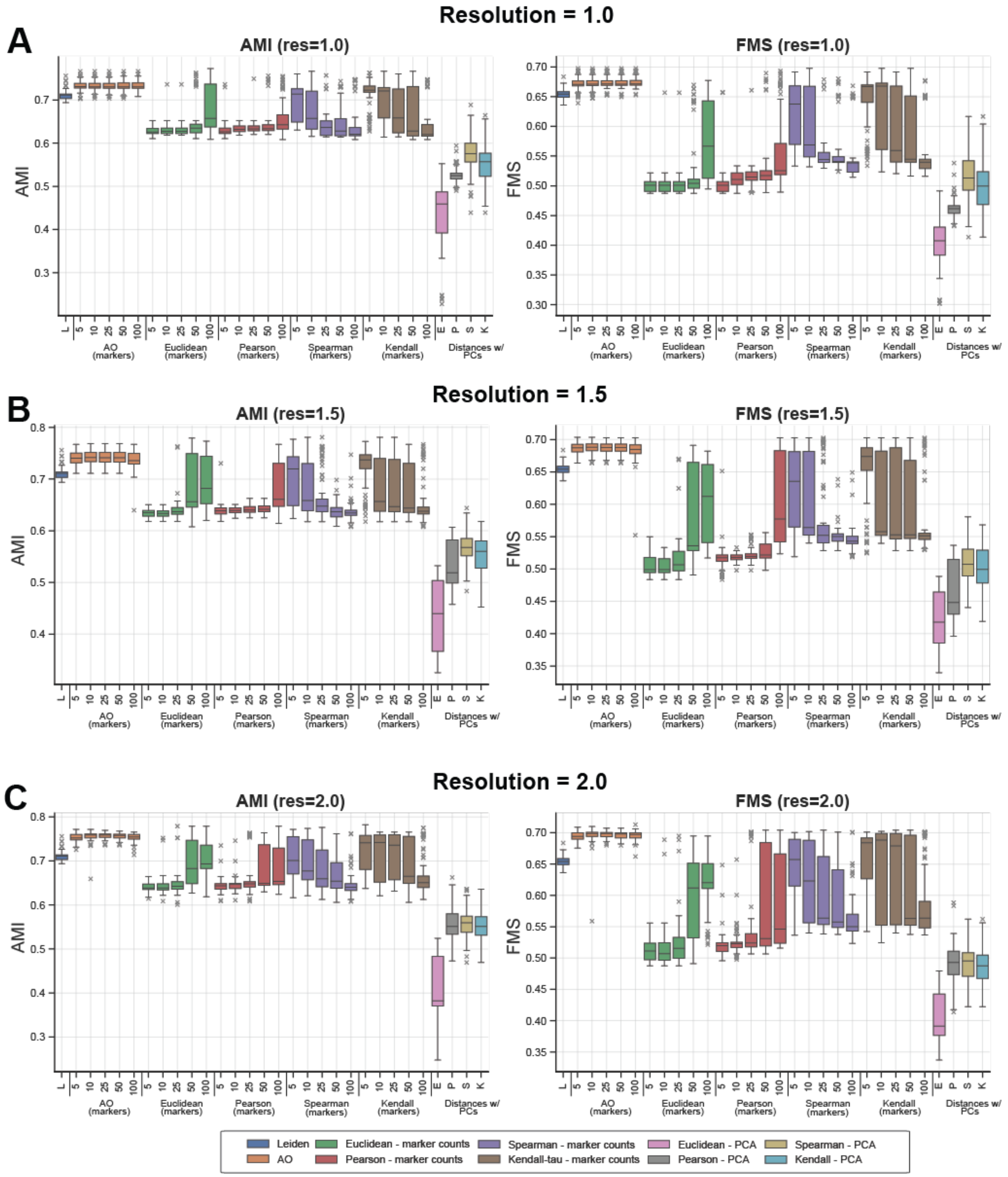

Appendix Figure S2. AMI and FMS scores in *Zhengmix8eq*.

(**A-C**) Adjusted Mutual Information (AMI) and Fowlkes-Mallows index (FMI) performance measures based on the concordance of ground truth labels with derived cell populations, across different metrics and number of marker genes used in the *Zhengmix8eq* dataset, with starting Leiden cluster resolution at 1.0 (**A**), 1.5 (**B**), and 2.0 (**C**).

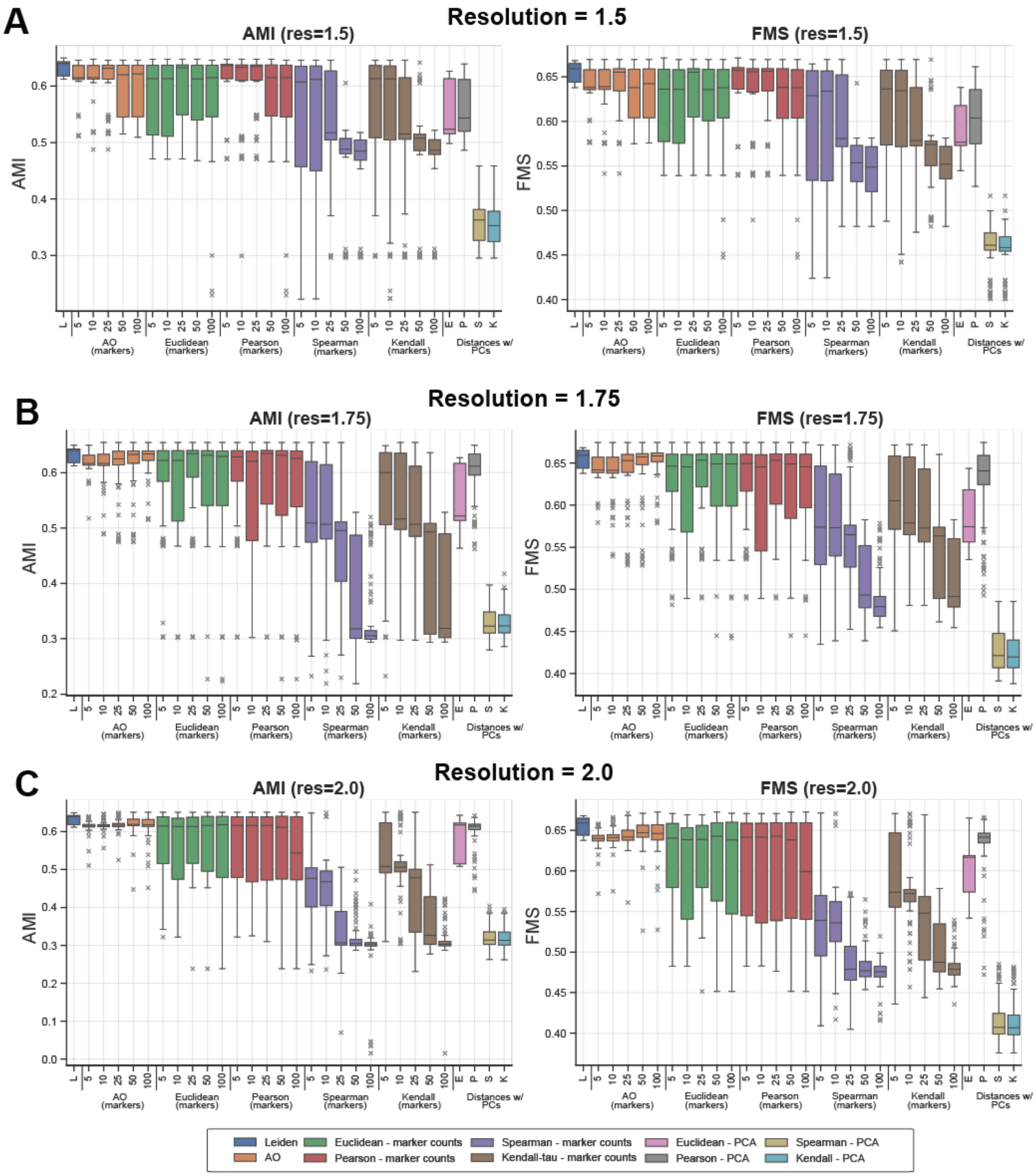

Appendix Figure S3. AMI and FMS of *Zhengmix8eq* T-cells.

Adjusted Mutual Information (AMI) and Fowlkes-Mallows index (FMI) performance measures based on the concordance of ground truth labels with derived cell populations, across different metrics and number of marker genes used in the T-cell subset of the *Zhengmix8eq* dataset, with starting Leiden cluster resolution at 1.5 (A), 1.75 (B), and 2.0 (C).

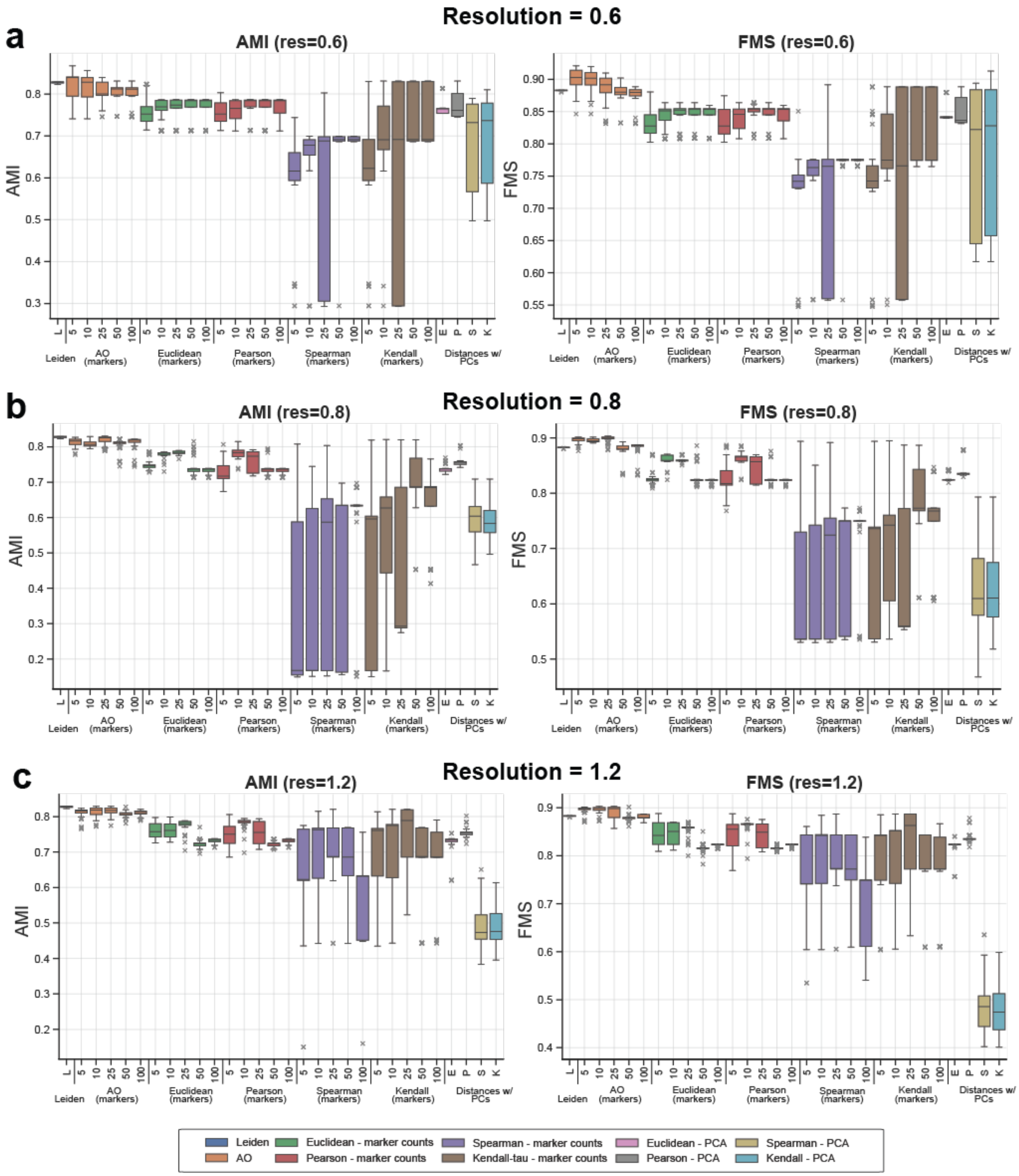

Appendix Figure S4. AMI and FMS of CBMCs.

Adjusted Mutual Information (AMI) and Fowlkes-Mallows index (FMI) performance measures based on the concordance of ground truth labels with derived cell populations, across different metrics and number of marker genes used in the CBMC dataset, with starting Leiden cluster resolution at 0.6 (A), 0.8 (B), and 1.2 (C).

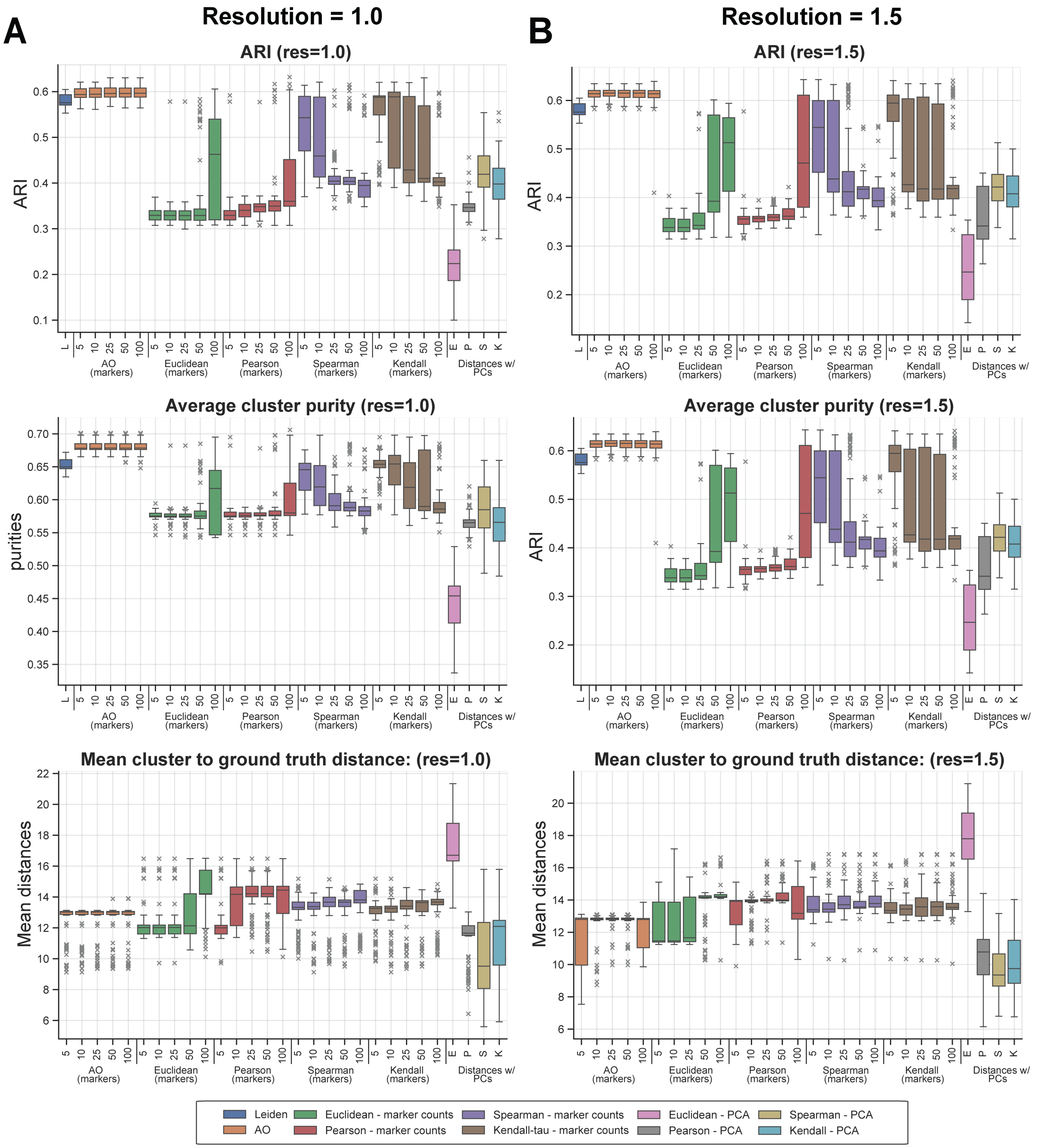

Appendix Figure S5. Performance metrics of *Zhengmix8eq* at other resolutions.
(**A-B**) ARI, average cluster purity, and mean distances between merged clusters and corresponding ground truth populations in the *Zhengmix8eq* dataset, for starting Leiden cluster resolutions 1.0 (**A**) and 1.5 (**B**).

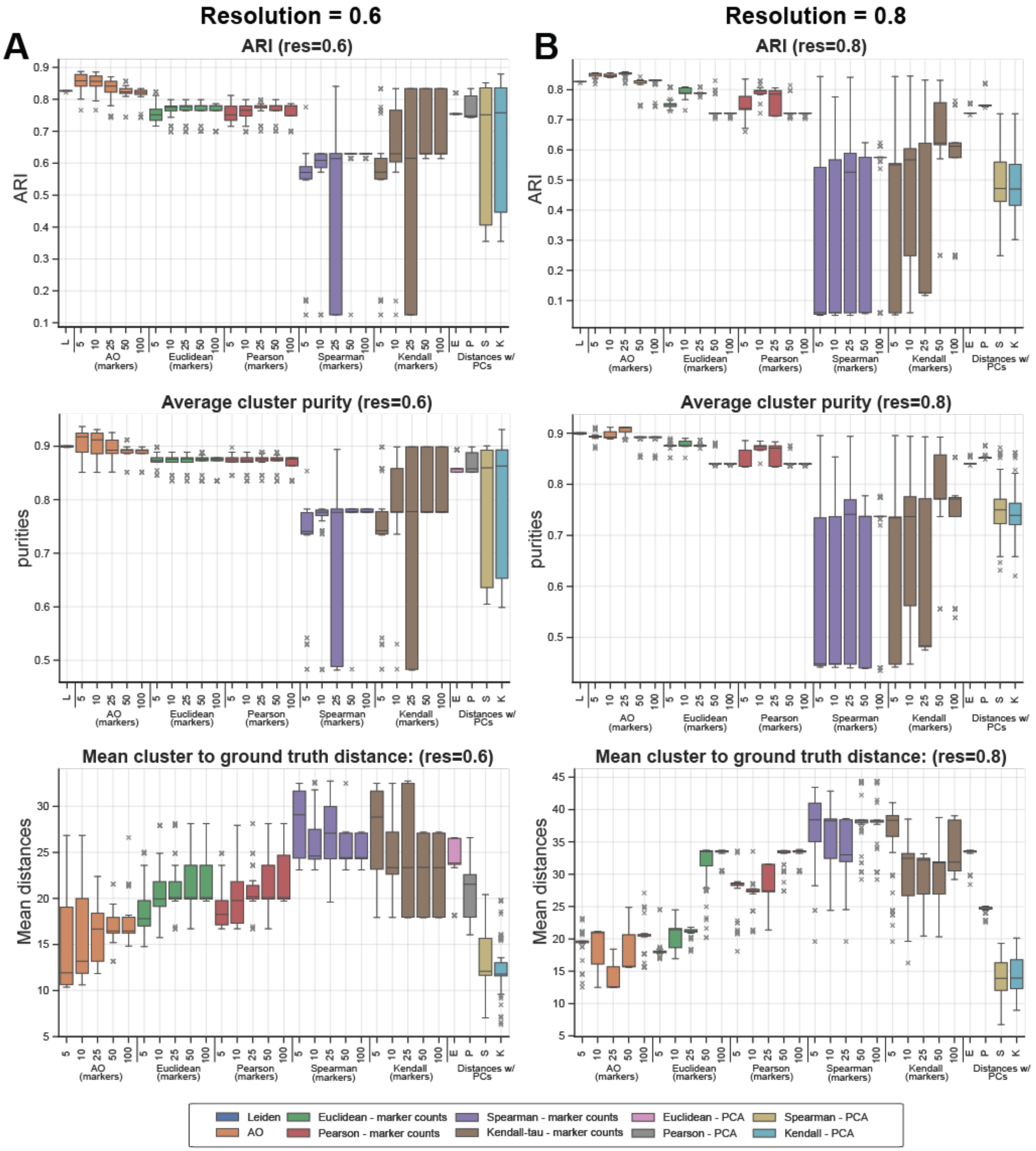

Appendix Figure S6. Performance metrics of CBMCs at other resolutions.

(**A-B**) ARI, average cluster purity, and mean distances between merged clusters and corresponding ground truth populations in the CBMC dataset, for starting Leiden cluster resolutions 0.6 (**A**) and 0.8 (**B**).

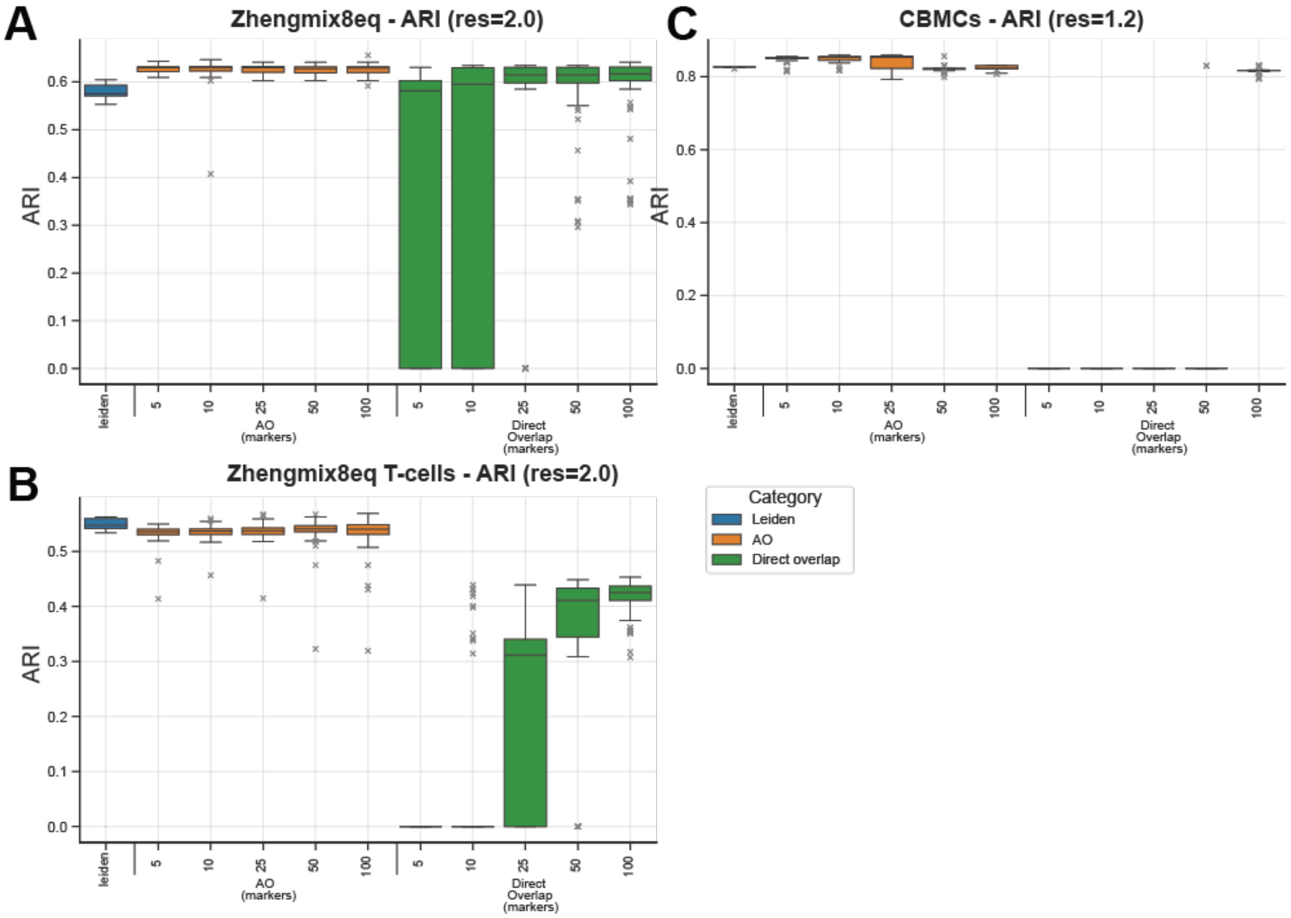

Appendix Figure S7. Baseline comparisons of performance metrics between clustering generated with AO, direct overlap, and using a fixed Leiden resolution.
(**A-C**) ARI when using finely tuned Leiden clusters without any refinement, and when using direct overlap of marker gene sets for hierarchical clusters in *Zhengmix8eq* (**A**), the T-cell subset in *Zhengmix8eq* (**B**), and CBMCs (**C**), in the highest starting Leiden resolution benchmarked in each dataset. Direct overlap shows poor performance when used with low numbers of cluster marker genes.

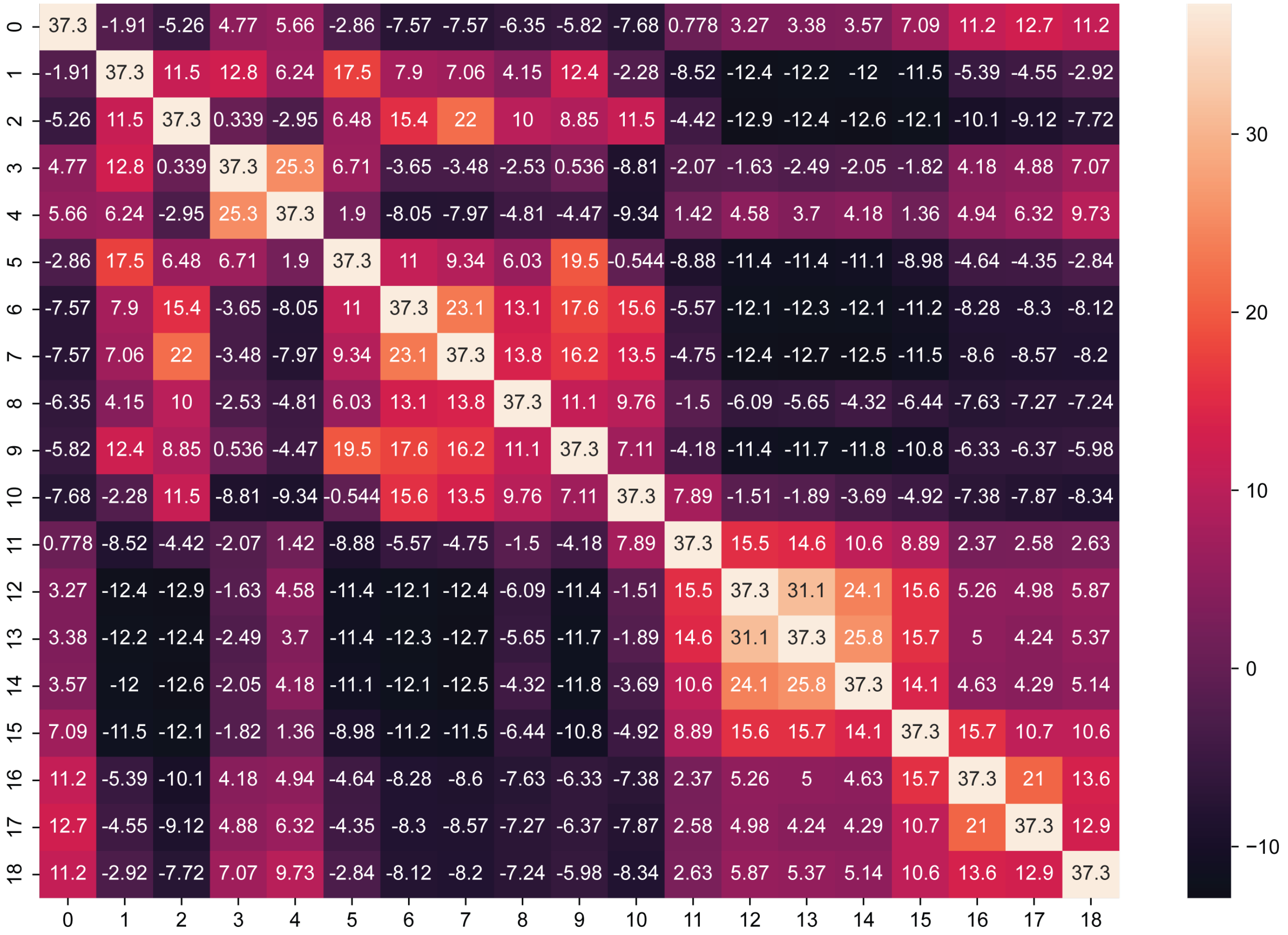

Appendix Figure S8. AO Heatmap of Leiden clusters in developing thymocytes. Pairwise AO scores between all 19 cell clusters obtained from Leiden clustering of developing thymocytes.

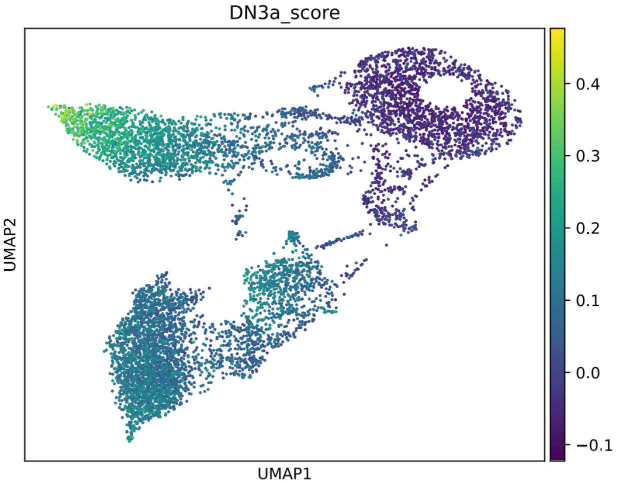

Appendix Figure S9. **Upregulation of DN3a- and DN3b-related genes in developing mice thymocytes**.

Cells were scored by expression of genes upregulated in purified wildtype DN3a and 3b cells, determined by a study from Vogel et al[35].

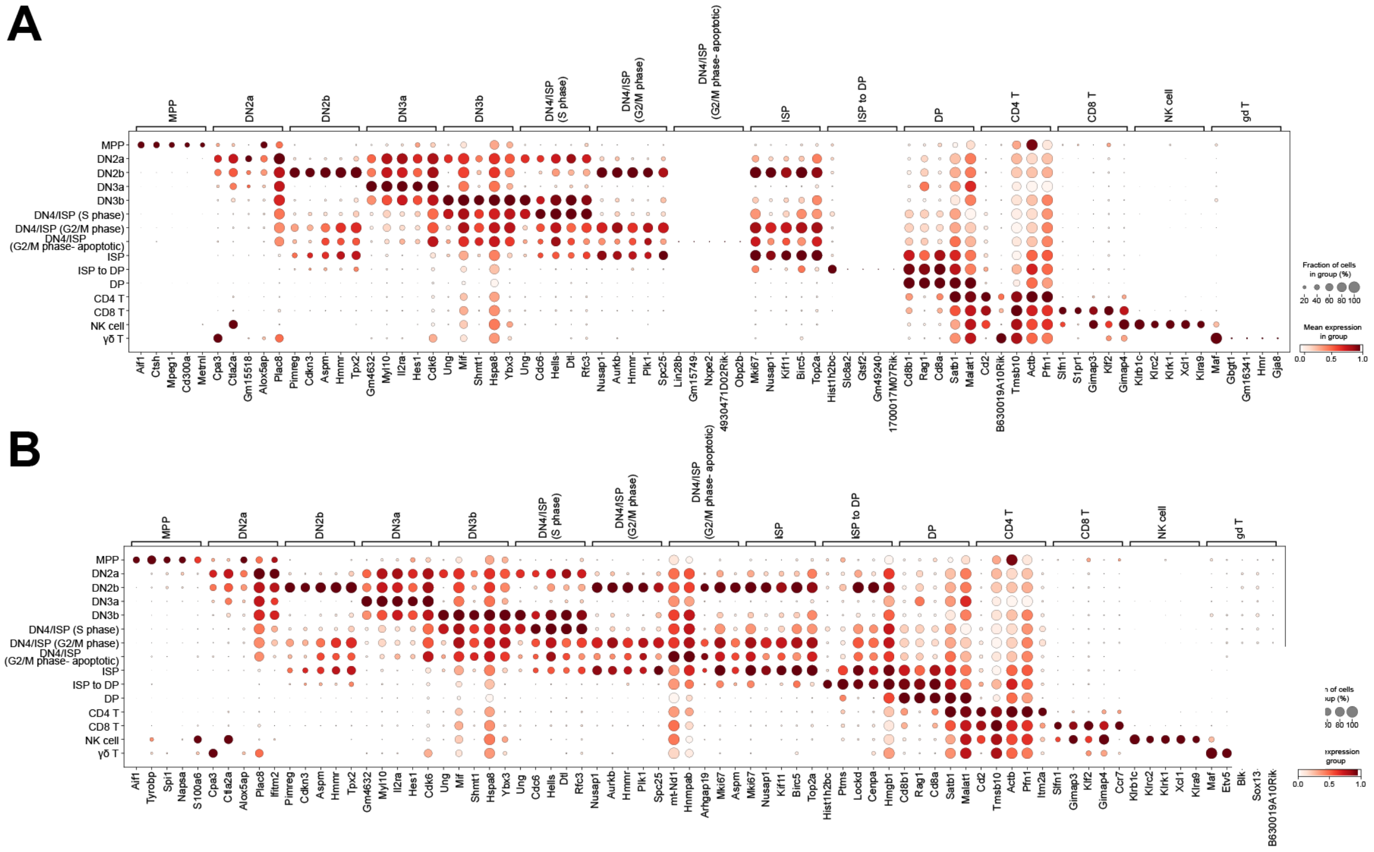

Appendix Figure S10. Top marker gene expression in each final annotated population in the thymus.

(**A**) The expression of top 5 marker genes for each annotated cell population in developing thymocytes.

(**B**) The expression of marker genes expressed in more than 50% of their respective cell population.

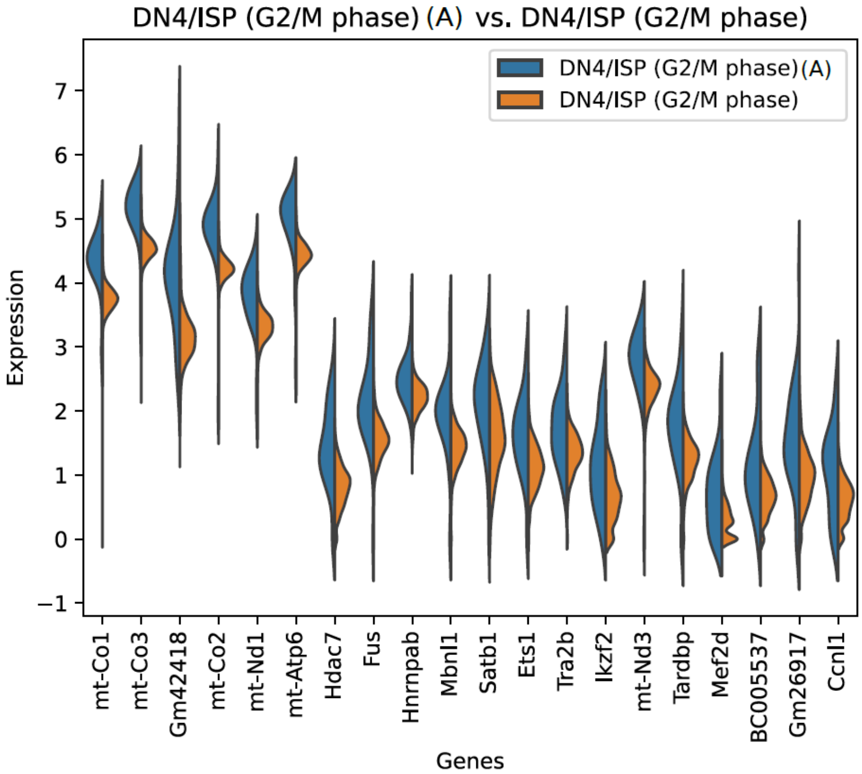

Appendix Figure S11. Pair-wise differential expression between apoptotic and normal DN4/ISP cells in G2/M phase.

Differentially expressed genes between these groups were determined with the Wilcoxon rank-sum test. Normalized expression of the top 20 genes upregulated in the apoptotic group are shown for both populations.

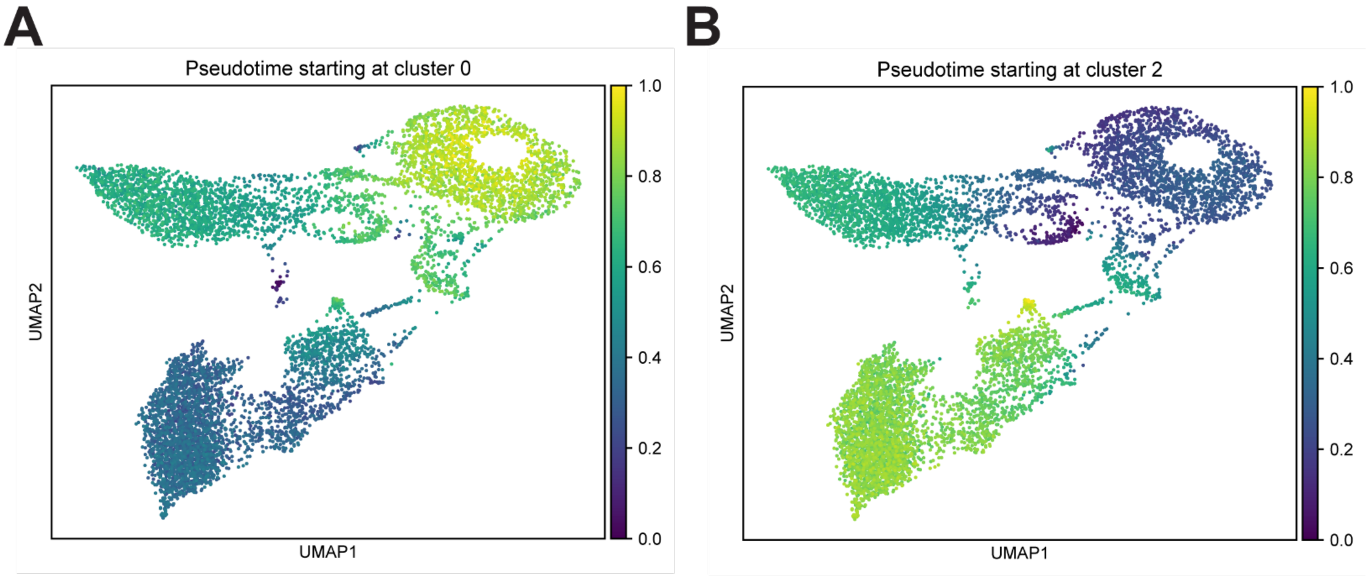

Appendix Figure S12. Diffusion pseudotime inferred on developing thymocytes.
(**A**) Among the group of cells that map to double negative thymocytes, when specifying Leiden cluster 0 as the starting point, the inferred trajectory closely matches our annotated stages of development.
(**B**) However, when setting cluster 2 as the start, two additional groups of cells, clusters 7 and 8, also share the same pseudotime value of 0.

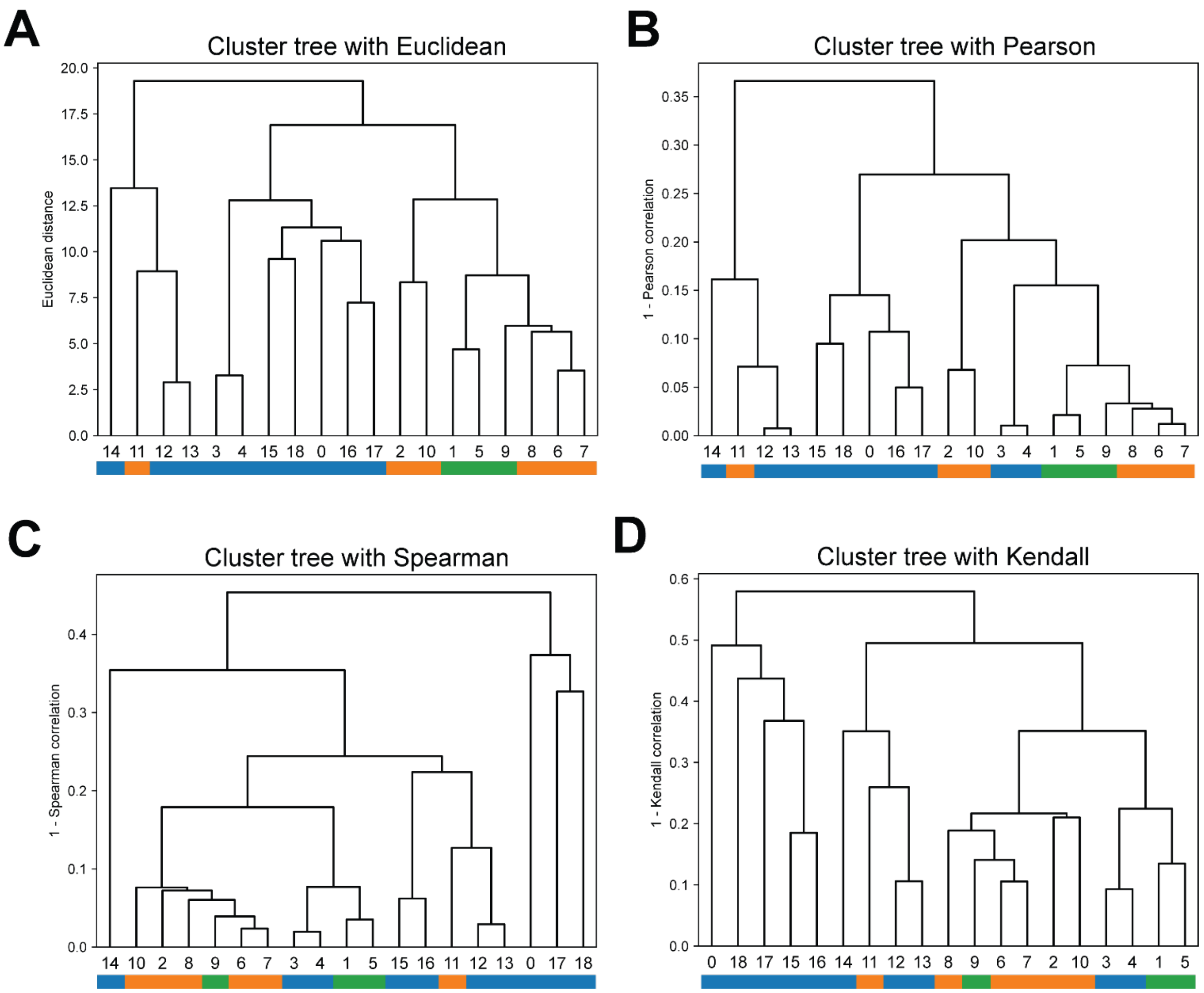

Appendix Figure S13. Hierarchical clustering of Leiden clusters in thymus data using Pearson, Spearman, and Kendall-Tau, and Euclidean distance.

(**A-D**) Cluster trees using Leiden clusters of developing thymocytes, using Euclidean (**A**), Pearson (**B**), Spearman (**C**), and Kendall-Tau metrics (**D**). All measures are calculated with the same set of top 25 marker genes in each Leiden cluster as previous analysis using AO. Grouping based on either cell cycle phase or neighboring stages of T-cell development is inconsistent.

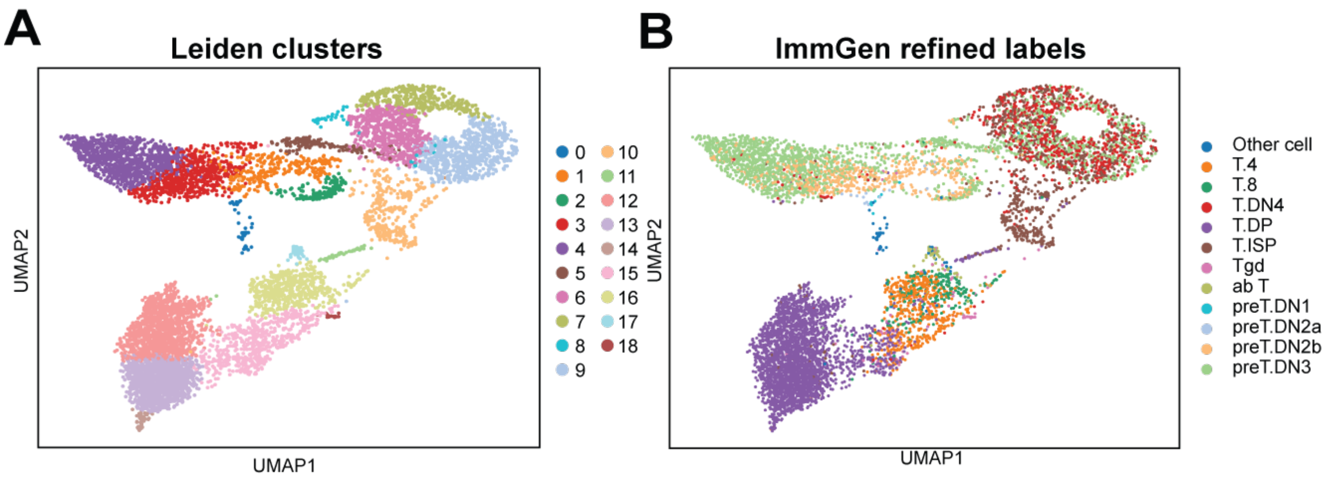

Appendix Figure S14. Automated annotation of thymus data using singleR.

(**A**) 19 cell clusters obtained from Leiden clustering of developing thymocytes.

(**B**) Cell labels obtained from automatic annotation of individual cells, using the singleR tool with bulk RNA-seq of purified thymocytes in ImmGen as a reference dataset.
