# Supplementary material for: CANTAO: guiding clustering and annotation in single-cell RNA sequencing using average overlap
Source: Mol Syst Biol. 2025 Dec 8;22(3):461–75. doi: 10.1038/s44320-025-00176-4 (PMC12954110; doi:10.1038/s44320-025-00176-4)
Supplement: Supplementary file 3 — Expanded View Figures [file 44320_2025_176_MOESM3_ESM.pdf]

Expanded View Figures

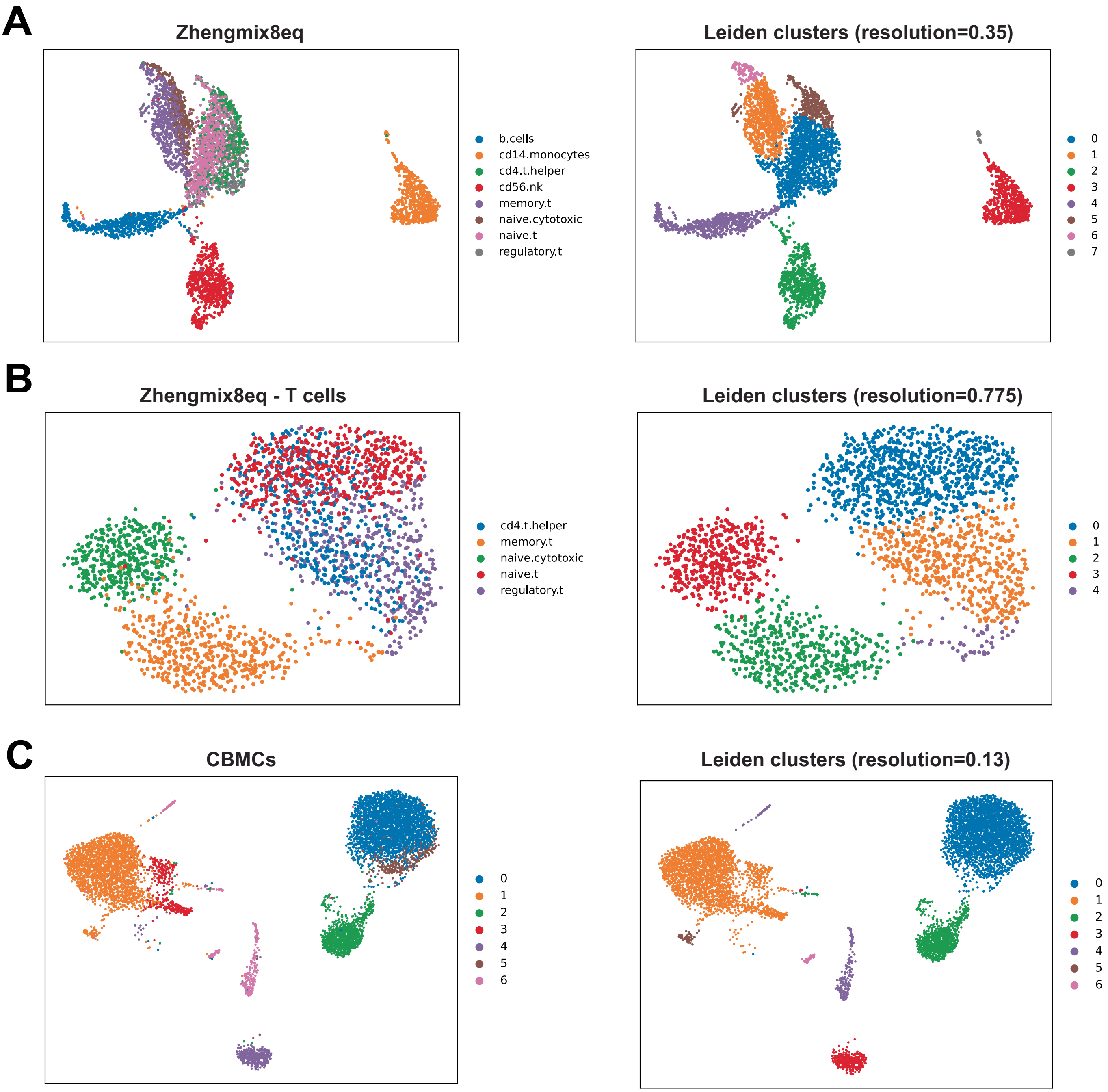

Figure EV1. UMAP projections of all datasets used in benchmarking with true labels and fine-tuned Leiden clusters.

(**A**) UMAP Projections for all cells in the *Zhengmix8eq* dataset. (**B**) UMAP projections of T cells in *Zhengmix8eq*. (**C**) UMAP projections of cells in CBMC dataset, produced from just RNA counts.

© The Author(s)

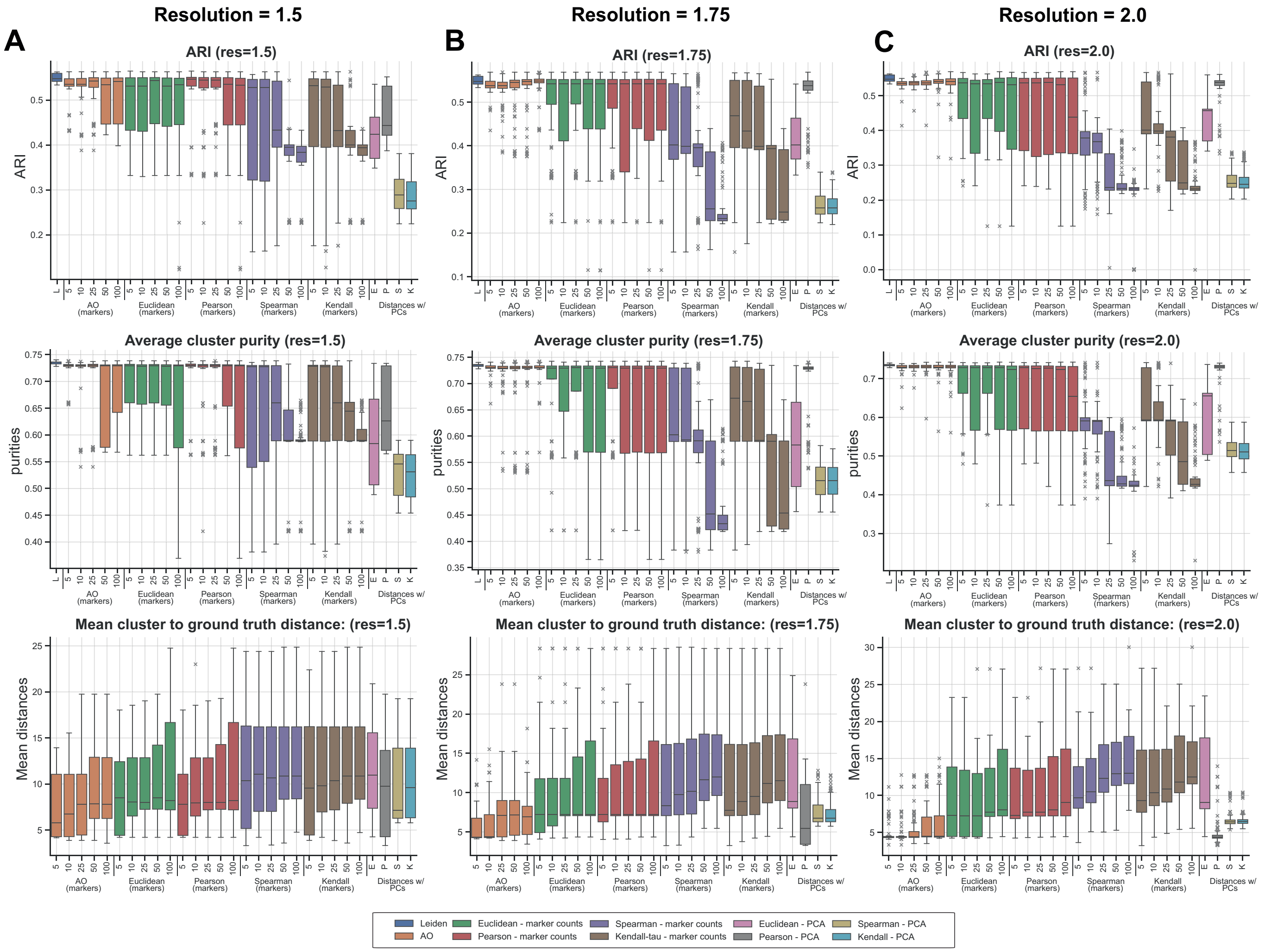

Figure EV2. Benchmarking average overlap versus other distance metrics used in hierarchical clustering of single-cell clusters for *Zhengmix8eq* T cells.

(**A–C**) Adjusted Rand Index (ARI), average cluster purity, and mean distances between inferred merged clusters and their corresponding ground truth populations, based on ground truth labels for 5 cell populations, across different metrics and number of marker genes used, in the T-cell subset of the *Zhengmix8eq* dataset. 5 final merged clusters were produced from initial Leiden clustering at resolutions 1.5 (**A**), 1.75 (**B**), and 2.0 (**C**). Data information: Hierarchical clustering was performed with each combination of metrics and/or number of cluster marker genes 100 times. In all boxplots (**A–C**), the midpoints represent median values. The bounds of the box correspond to the 25th and 75th percentile values (Q1 and Q3, respectively). The whiskers of the plots extend to points that lie within 1.5 IQRs of the lower and upper quartiles Q1 and Q3, where IQR = Q3 – Q1 is the interquartile range. Observations that fall outside this range are displayed independently.

 © The Author(s)

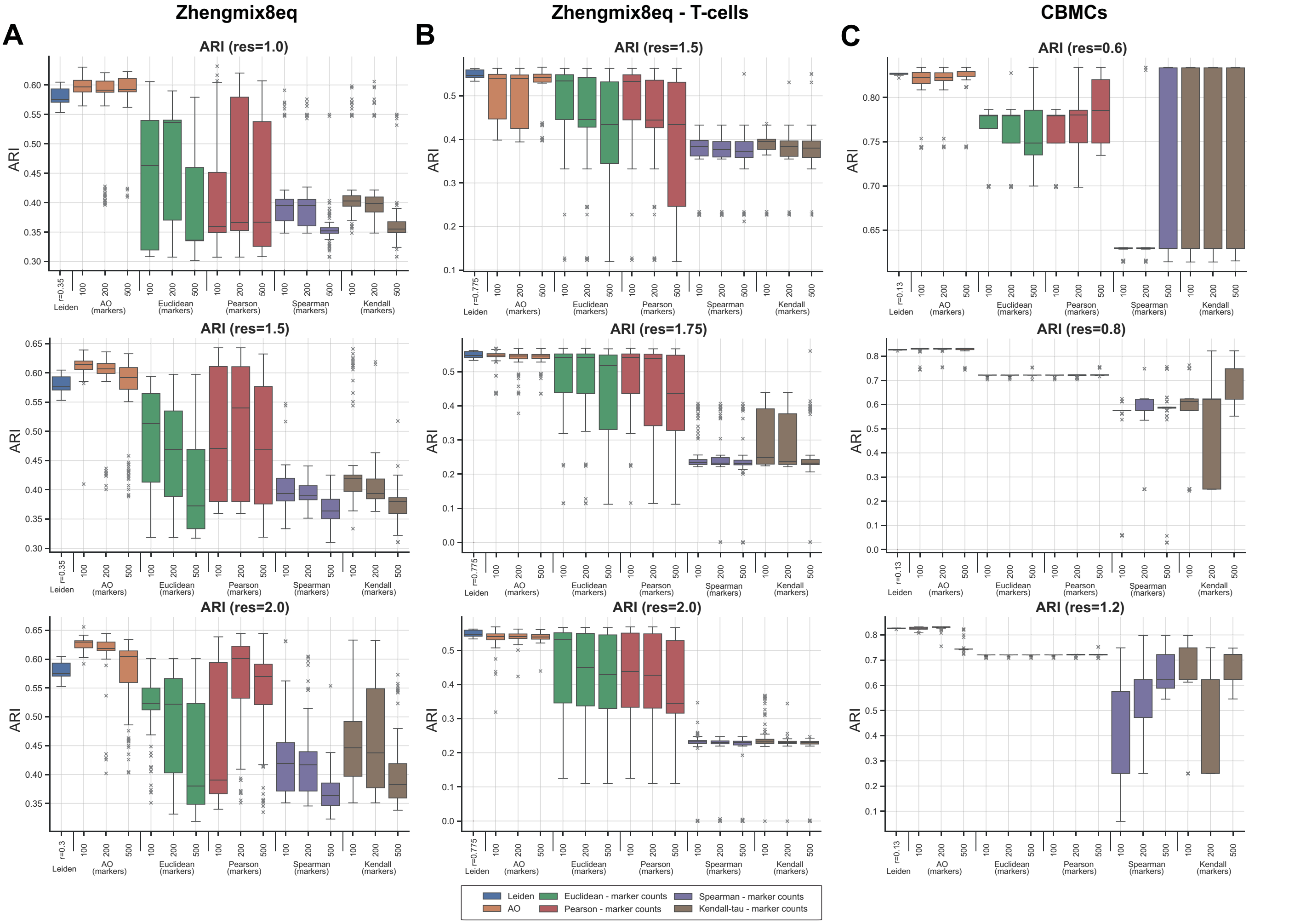

Figure EV3. ARI in *Zhengmix8eq*, its T-cell subset, and CBMCs when utilizing a high number of cluster marker genes.

(**A–C**) ARI of all benchmarks in *Zhengmix8eq* (**A**), the T-cell subset in *Zhengmix8eq* (**B**), and CBMCs (**C**), for all starting Leiden cluster resolutions tested, when defining marker gene sets of length 100, 200, and 500. For AO, there is no noticeable performance gain from using a higher number of marker genes. Data information: Hierarchical clustering was performed with each combination of metrics and/or number of cluster marker genes 100 times. In all boxplots (**A–C**), the midpoints represent median values. The bounds of the box correspond to the 25th and 75th percentile values (Q1 and Q3, respectively). The whiskers of the plots extend to points that lie within 1.5 IQRs of the lower and upper quartiles Q1 and Q3, where IQR = Q3 – Q1 is the interquartile range. Observations that fall outside this range are displayed independently.

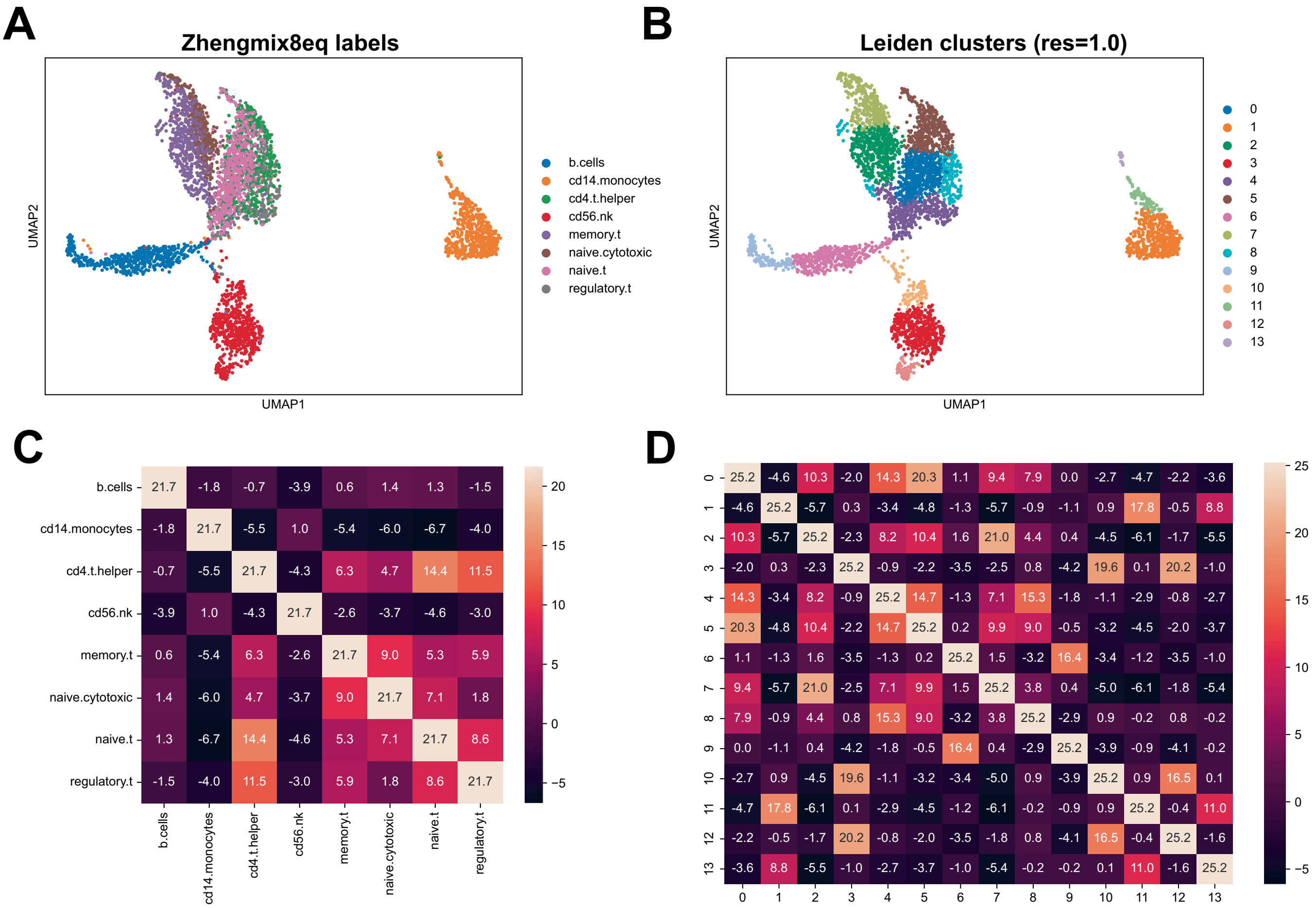

Figure EV4. Interpreting significance of AO similarity in an example partitioning of the *Zhengmix8eq* dataset.

(**A**) UMAP of the *Zhengmix8eq* dataset with true cell labels. (**B**) A sample Leiden clustering (resolution = 1.0) of the *Zhengmix8eq* dataset projected on the same UMAP. (**C**) Pairwise AO scores between true cell populations, converted to z-scores. AO was calculated on rankings of a global marker gene set composed of the top 25 differentially expressed genes in each population. (**D**) Pairwise AO scores between unsupervised Leiden clusters, converted to z-scores, similar to (**C**).

 © The Author(s)

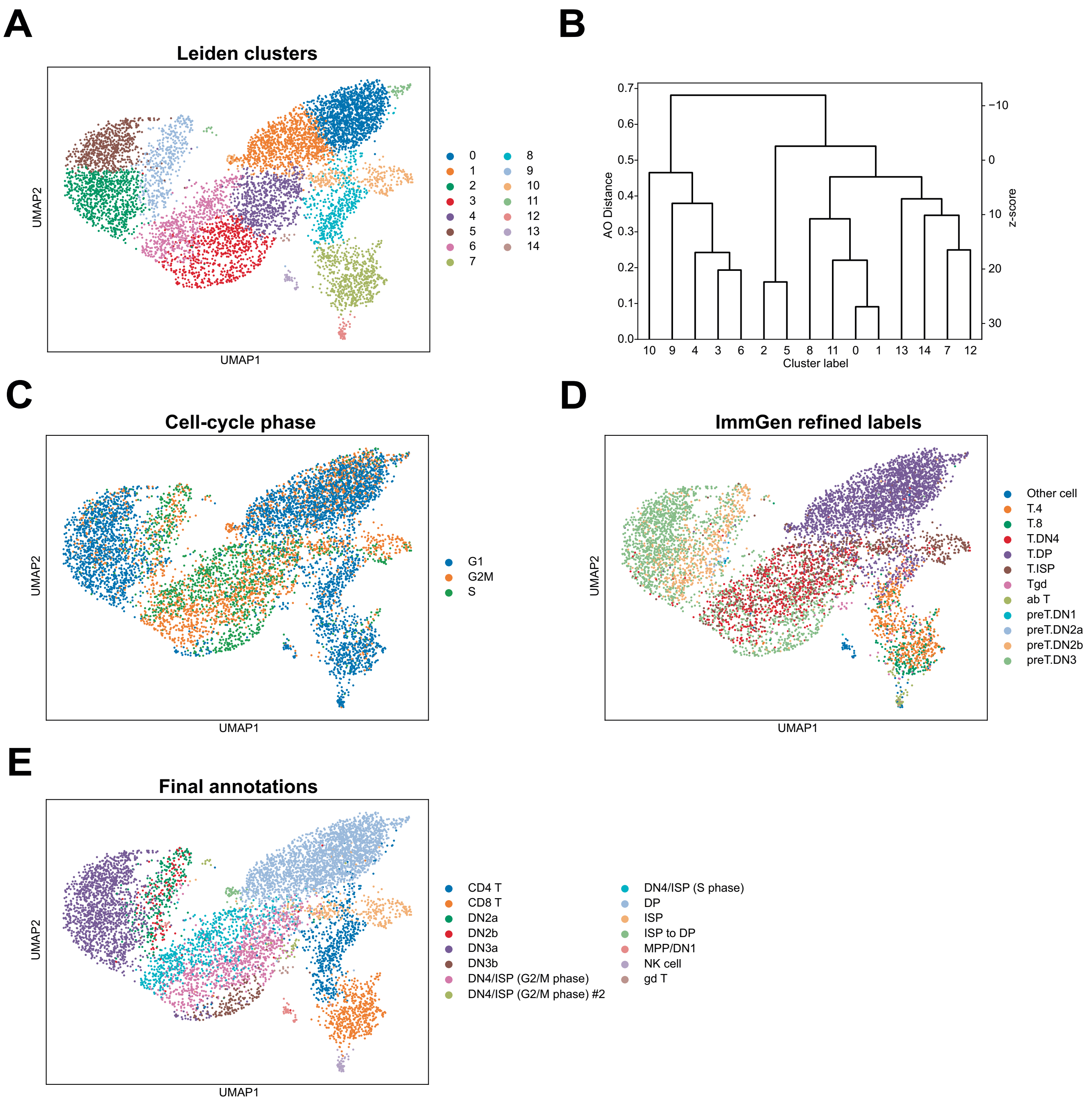

Figure EV5. The effect of regressing out cell cycle effects from thymus data.

(**A**) UMAP plot of Leiden clustering of thymus data when regressing out cell cycle effects, resulting in 15 populations. (**B**) AO tree of Leiden clusters shown in (**A**). (**C**) Annotated cell cycle phases for each cell, projected on the UMAP produced after regressing out cell cycle. (**D**) Annotations of individual cells, using the singleR tool with bulk RNA-seq of purified thymocytes in ImmGen as a reference dataset, overlaid on the UMAP produced after regressing out cell cycle. (**E**) Final annotations of the original analysis of thymus data, overlaid on the UMAP produced after regressing out cell cycle.

© The Author(s)
